# Inhibitory Receptor Signaling Destabilizes Immunological Synapse Formation in Primary NK Cells

**DOI:** 10.3389/fimmu.2013.00410

**Published:** 2013-11-27

**Authors:** Thushara P. Abeyweera, Molly Kaissar, Morgan Huse

**Affiliations:** ^1^Immunology Program, Memorial Sloan-Kettering Cancer Center, New York, NY, USA

**Keywords:** NK cell, signal transduction, immunological synapses, ITIM, imaging

## Abstract

Upon engagement of their cognate class I major histocompatibility complex ligands, receptors containing immunotyrosine-based inhibitory motifs (ITIMs) transduce signals that block cytolytic and inflammatory responses. In this manner, ITIM-coupled receptors play a crucial role in maintaining natural killer (NK) cell tolerance toward normal, healthy tissue. A number of studies, mostly using immortalized NK cell lines, have demonstrated that ITIM signaling functions by disrupting the cytolytic immunological synapse formed between an NK cell and its target. However, more recent imaging experiments using primary NK cells have suggested that inhibitory receptor engagement does not antagonize contact formation, casting doubt on the hypothesis that ITIM signals destabilize the synapse. To resolve this issue, we analyzed primary NK cell activation and contact formation on supported lipid bilayers containing controlled combinations of activating and inhibitory ligands. Under these conditions, we observed that ITIM signaling clearly inhibited adhesion, cell arrest, and calcium influx, three hallmarks of synapse formation. These results are consistent with previous reports showing that inhibitory receptors deliver a “reverse stop” signal, and confirm that ITIM signaling functions at least in part by destabilizing cytolytic synapse formation.

## Introduction

Natural killer (NK) lymphocytes play an important role in anti-viral and anti-tumor responses by specifically eliminating cells that bear signs of infection or transformation. Target cell recognition triggers the formation of a stereotyped junction between the NK cell and the target known as an immunological synapse (IS) (1, 2). This is followed by directional release of cytolytic perforins and granzymes into the synaptic space, leading to target cell death by apoptosis. By mediating adhesion and focusing secretion in this manner, the IS promotes target cell killing while limiting damage to surrounding healthy tissue.

Natural killer cell cytotoxicity is governed by a number of distinct activating and inhibitory cell-surface receptors (3, 4). Activating receptors induce IS formation, target cell killing, and the release of inflammatory cytokines such as interferon-γ, while inhibitory receptors transduce signals that block these activating responses. Activating receptors are quite structurally diverse, and bind to ligands that are indicative of infection, transformation, or immune targeting. The C-type lectin NKG2D, for example, recognizes a set of proteins (including the MIC and ULBP families) that are upregulated in response to cellular stress. CD16, by contrast, is a low affinity Fc receptor that enables engagement of antibody-coated targets. Inhibitory NK receptors, for their part, almost exclusively recognize class I major histocompatibility complex (MHC), which is highly expressed in normal, healthy tissue. This leads to the phosphorylation of immunotyrosine-based inhibitory motifs (ITIMs) located in the cytoplasmic tail of the receptor. Phosphorylated ITIMs recruit the tyrosine phosphatases SHP-1 and -2, which are thought to dephosphorylate signaling proteins required for NK cell activation.

Precisely how inhibitory receptor engagement blocks activating responses in NK cells remains an area of intense interest. SHP-1 has been shown to dephosphorylate Vav-1 (5), a large scaffolding protein and guanine nucleotide exchange factor involved in multiple activating pathways. ITIM-receptor signaling has also been linked to the phosphorylation of the adaptor protein Crk and its dissociation from activating signaling complexes (6, 7). Translating these biochemical events into a cellular response, however, has been challenging. To address this deficiency, a number of groups have employed videomicroscopy approaches in which individual NK cells are imaged on surfaces containing defined mixtures of activating and inhibitory NK receptor ligands (6, 8, 9). If sufficient activating ligand is present, NK cells form stable, symmetric contacts on these surfaces that bear the structural hallmarks of an IS. Because these contacts are positioned at the cell-surface interface in an orientation perpendicular to the axis of illumination, it is possible to image them using high-resolution modalities such as total internal reflection fluorescence (TIRF) microscopy.

To facilitate day-to-day experimentation, most studies using this approach have employed human cell lines such as NKL, which was derived from an NK cell leukemia. Initial efforts focused on the effects of NKG2A, a C-type lectin that binds to the non-classical MHC HLA-E. NKG2A engagement strongly inhibited IS formation on stimulatory glass surfaces, inducing instead an active migratory phenotype (9). These results suggested that NKG2A delivers a “reverse stop” signal that antagonizes stable contact with the target cell. Subsequently, our lab analyzed inhibitory signaling from the killer immunoglobulin receptor KIR2DL2 using a supported lipid bilayer system that allows free diffusion of activating and inhibitory ligands (8). Stimulation of KIR2DL2 with its cognate ligand HLA-Cw3 essentially blocked IS formation in these experiments. Using a photostimulation approach, we also demonstrated that KIR2DL2 signaling induced the retraction of preexisting synapses. These results lent further support to the idea that inhibitory receptor engagement destabilizes the IS. They were also consistent with previous studies indicating that ITIM signaling disrupts NK cell-target cell adhesion (10, 11).

More recently, the stimulatory bilayer approach was employed to examine inhibitory signaling in primary human NK cells (6). Surprisingly, contacts were observed on bilayers containing HLA-E, implying that NKG2A signaling does not disrupt IS formation. The authors of this study attributed discrepancies between their data and previous results to differences between primary NK cells and the NKL cell line. However, the relative strength of the observed inhibitory contacts was not assessed relative to activating synapses, and the relative motility of NK cells on activating and inhibitory bilayers was not quantified. To address these issues, we have profiled the activation status and migratory behavior of primary human NK cells on both activating and inhibitory bilayers. Our results indicate that ITIM-receptors do indeed destabilize cytolytic IS formation and promote migration by delivering a reverse stop signal.

## Results

### Activation and inhibition of primary NK cells on lipid bilayers

Supported lipid bilayers containing purified activating and inhibitory ligands are increasingly used as target cell proxies in imaging-based studies of NK cell signaling. A number of strategies exist for incorporating ligands into the bilayer. For our experiments, we used a 1:10 mixture of 1,2-dioleoyl-*sn*-glycero-3-phosphocholine (DOPC) and the biotinylated lipid 1,2-dioleoyl-*sn*-glycero-3-phosphoethanolamine-*N*-(cap biotinyl). Treatment of these bilayers with streptavidin followed by controlled amounts of biotinylated NK receptor ligands results in orientated incorporation of these ligands into the membrane.

**Figure 1:**
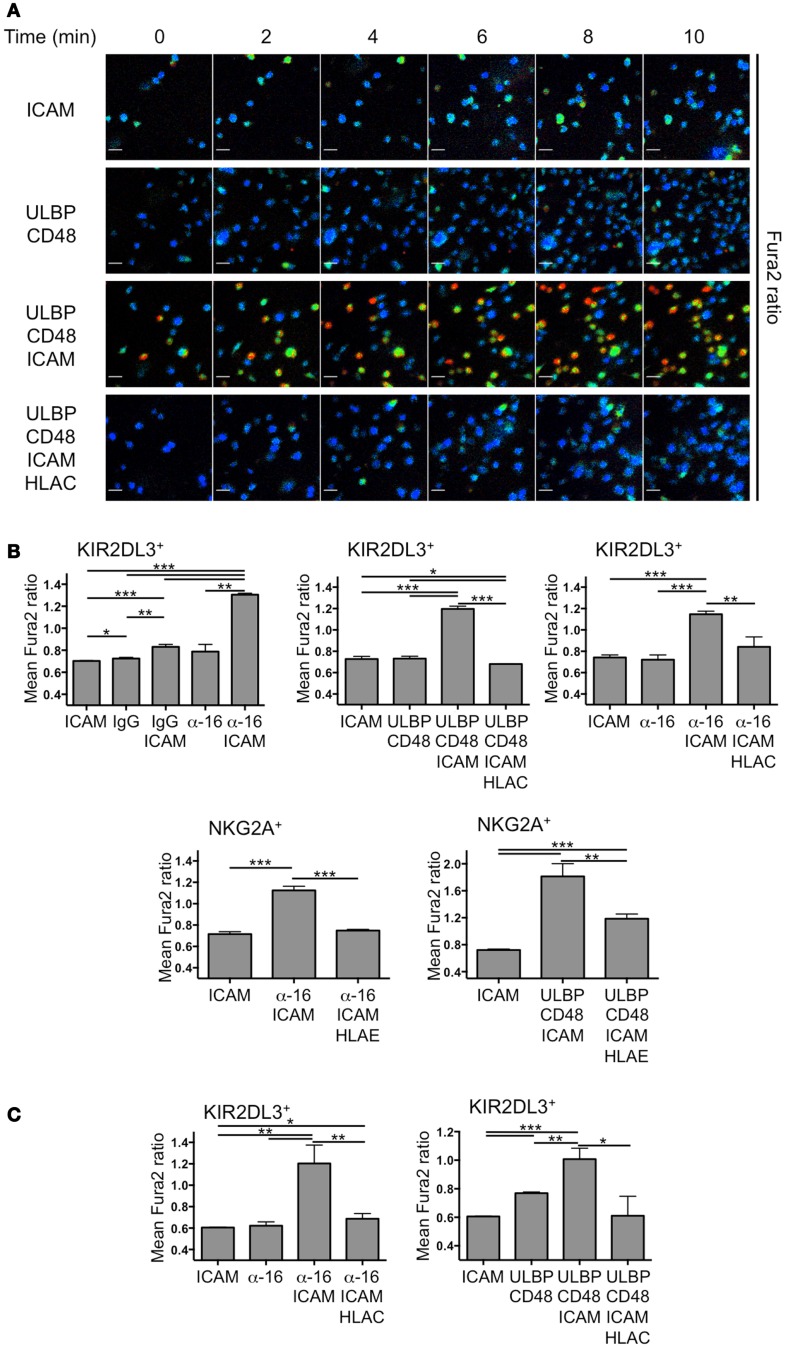
**Primary NK cell activation on stimulatory lipid bilayers**. IL-2-cultured and resting primary human NK cells were sorted into KIR2DL3^+^ or NKG2A^+^ populations, loaded with Fura2-AM and imaged on supported lipid bilayers. **(A)** Time-lapse montages from a representative experiment showing single cell Ca^2+^ responses from KIR2DL3^+^ cells on bilayers containing the indicated activating and inhibitory NK receptor ligands. Fura2 ratio is pseudocolored with cooler and warmer colors indicating low and high intracellular Ca^2+^ concentration, respectively. **(B,C)** Ca^2+^ influx was quantified for IL-2-cultured **(B)** or resting **(C)** KIR2DL3^+^ or NKG2A^+^ cells as indicated by calculating the average Fura-2 ratio for all cells in the imaging field during the plateau phase of the global response (see Materials and Methods). Error bars denote standard error of the mean (SEM). *P*-values were calculated using Student’s *t*-test, with ***P < 0.001, **P < 0.01, and *P ≤ 0.05. HLAC = HLA-Cw3; HLA-E = HLA-E; ULBP = ULBP3; α-16 = anti-CD16.

We obtained primary human NK cells expressing NKG2A or KIR2DL3 from peripheral blood of a group A haplotype donor. To establish the stimulus requirements for the activation and inhibition of these cells, we monitored calcium (Ca^2+^) responses in highly pure ( >95% CD3^−^CD56^+^) NK cell preparations using the Ca^2+^ sensitive dye Fura-2 (Figure 1A). Ca^2+^ flux is a common feature of activating NK receptor signaling pathways and is required for cytolytic degranulation responses (3, 12–14). Hence, it serves as a reliable index of activation status at a single cell level. NK cells were stimulated by simultaneous engagement of NKG2D and the SLAM family receptor 2B4 with a mixture of their respective cognate ligands, ULBP3 and CD48. We also activated NK cells through CD16 using an anti-CD16 antibody, which we found to be more stimulatory than IgG, the cognate CD16 ligand (Figure 1B). Activating ligands were presented to NK cells both in the presence and the absence of the adhesion molecule ICAM-1, which binds to the α_L_β_2_ integrin LFA-1. In this manner, we were able to assess the importance of integrin coengagement for NK cell activation. Finally, purified HLA-Cw3 and HLA-E were used to inhibit activating signals in KIR2DL3^+^ and NKG2A^+^ NK cells, respectively.

Ca^2+^ responses were visualized by ratiometric Fura-2 imaging at low magnification, enabling quantification over many cells. Activation at a population level was defined as elevation of intracellular Ca^2+^ levels above background during the sustained phase of the response (between 8 and 16 min after the start of the experiment). Remarkably, coengagement of activating receptors together with LFA-1 was a prerequisite for NK cell activation under these conditions (Figure 1B). Neither anti-CD16 nor the combination of ULBP3 and CD48 induced appreciable activation in the absence of ICAM-1. Similarly, ICAM-1 alone was incapable of driving Ca^2+^ flux without activating receptor stimulation. Addition of HLA-Cw3 and HLA-E robustly inhibited activating responses in KIR2DL3^+^ and NKG2A^+^ NK cells, respectively, often restoring intracellular Ca^2+^ to background levels (Figure 1B). These data were consistent with previous reports demonstrating the potential of ITIM-derived signals to override activating pathways at a very early stage (15–19). Although we initially used IL-2-cultured NK cells for these experiments, we observed a similar pattern of results using resting NK cells (Figure 1C); ICAM-1 was required for robust Ca^2+^ influx, and this response was largely blocked by inhibitory receptor engagement. We conclude that LFA-1 stimulation is crucial for primary NK cell activation on lipid bilayers, and that this activation is reversed by inhibitory receptor engagement.

### ITIM-receptors deliver a reverse stop signal in IL-2-cultured NK cells

Stable cytolytic IS formation is associated with an arrest in cell motility, and signals from inhibitory receptors have been proposed to overcome this arrest by delivering a reverse stop signal (9). To assess the validity of this model in our system, we quantified the movement of KIR2DL3^+^ and NKG2A^+^ primary IL-2-cultured NK cells on lipid bilayers containing various combinations of activating and inhibitory ligands. Cells were stained with the membrane dye PKH26 and imaged by TIRF microcopy, which enabled us to focus on the behavior of cells in contact with the bilayer.

**Figure 2:**
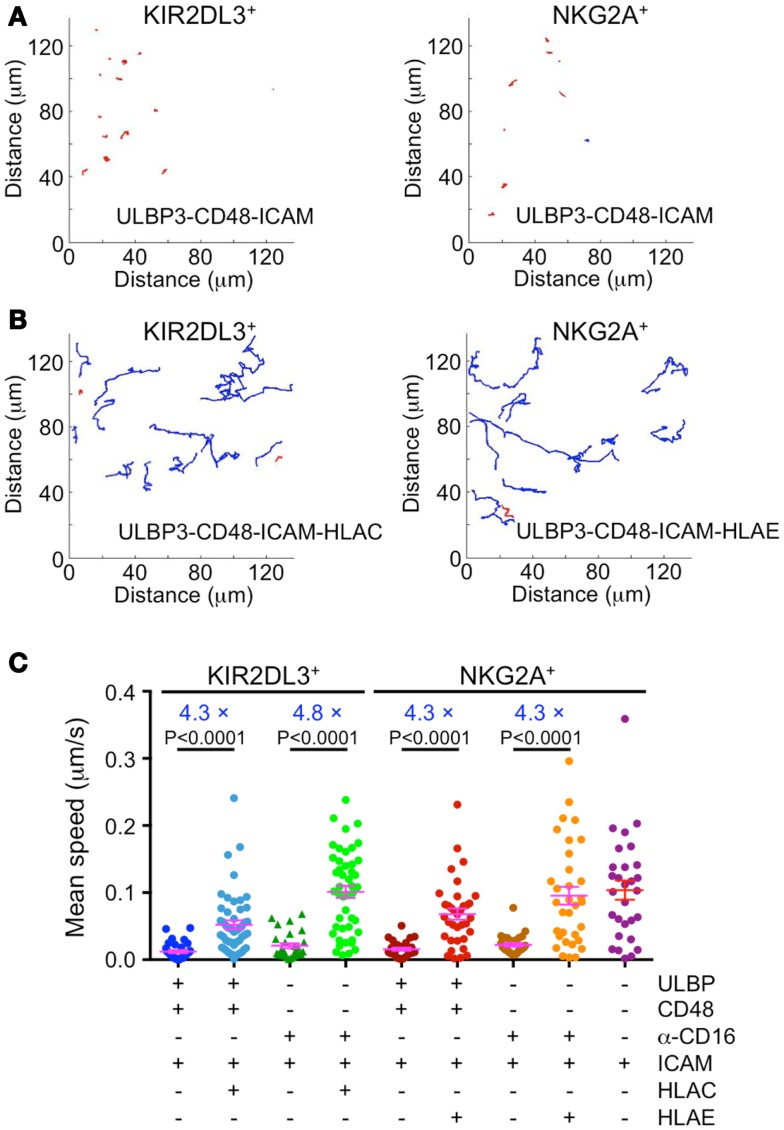
**Immunotyrosine-based inhibitory motif-receptor signaling induces a reverse stop signal**. IL-2-cultured primary human NK cells were labeled with the membrane dye PKH26 and imaged by TIRF microscopy on supported lipid bilayers containing activating and inhibitory NK receptor ligands. **(A,B)** Representative trajectories for KIR2DL3^+^ or NKG2A^+^ NK cells on bilayers containing either activating ligands alone **(A)** or mixtures of activating and inhibitory ligands **(B)**. Blue trajectories denote average speed ≥30 nm/s, while red trajectories denote average speed <30 nm/s. **(C)** Average speed of KIR2DL3^+^ or NKG2A^+^ NK cells on bilayers containing the indicated activating and inhibitory ligands (*n* ≥ 28 cells per condition). Factors relating average speed in the presence or absence of inhibitory ligand for each activating condition are shown in blue. Magenta lines and error bars indicate mean and SEM. *P*-values were calculated using the Mann–Whitney test.

Single cell tracking analysis revealed that NK cell activation and inhibition were associated with dramatic differences in cell motility (Figures 2A,B). Under conditions of simultaneous activating receptor and LFA-1 stimulation, cells formed stable, largely stationary contacts characterized by a mean instantaneous velocity between 10 and 20 nm/s (Figures 2A,C). By contrast, coengagement of either KIR2DL3 or NKG2A increased mean velocity to between 50 and 100 nm/s, similar to the motility exhibited by NK cells on bilayers containing ICAM alone (Figures 2B,C). ITIM-receptor signaling appeared to have a weaker effect on cells activated via NKG2D and 2B4 than on cells activated via CD16 (Figure 2C). However, activation through NKG2D/2B4 also resulted in more pronounced cell arrest in our hands. Indeed, after accounting for differences in the degree of activation, we found that inhibitory receptor stimulation induced a four- to fivefold increase in motility under all stimulus conditions (Figure 2C). Taken together, these data demonstrate that engagement of KIR2DL3 or NKG2A relieves activation-induced arrest in primary NK cells, consistent with the model whereby ITIM-receptors deliver a reverse stop signal.

### ITIM-receptor signaling reduces adhesion in resting NK cells

**Figure 3:**
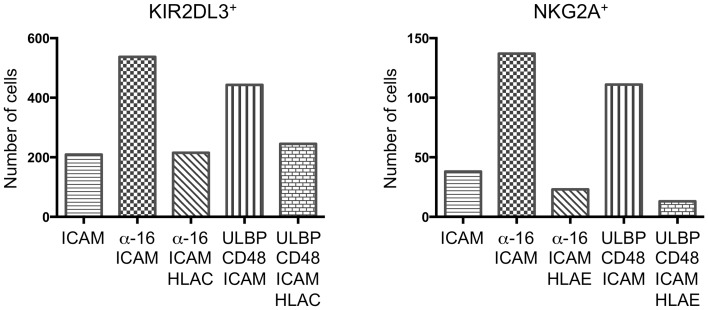
**Immunotyrosine-based inhibitory motif-receptor signaling inhibits attachment in resting NK cells**. Resting KIR2DL3^+^ (left) or NKG2A^+^ (right) NK cells were stained with PKH26 and plated on bilayers containing the indicated activating and inhibitory NK receptor ligands. After washing, attached cells were fixed and counted. A representative experiment is shown.

Next, we sought to extend our studies of synaptic stability to resting NK cells. However, under conditions where IL-2-cultured NK cells were highly dynamic (e.g., ICAM-1 alone), resting NK cells exhibited little to no motion. This made it difficult to use motility to assess the quality of synapses formed by these cells. We did notice however, that fewer cells became immobilized on bilayers containing inhibitory ligands. Accordingly, we implemented an imaging-based adhesion assay to quantify the number of cells making strong contact under both activating and inhibitory conditions. Cells were incubated with bilayers for 10 min, followed by a round of washing with PBS. Then, the remaining cells in contact with the bilayer were quantified by epifluorescence imaging at low resolution (see Materials and Methods). Both KIR2DL3^+^ and NKG2A^+^ resting NK cells exhibited basal adhesion on bilayers containing ICAM-1 alone (Figure 3). Addition of activating receptor ligands enhanced this adhesion two- to threefold, indicative of activating IS formation. Importantly, stimulation of either KIR2DL3 or NKG2A reduced adhesiveness to background levels (Figure 3). Indeed, often fewer cells were observed on bilayers containing combined activating and inhibitory ligands than on bilayers presenting ICAM-1 alone. We conclude that ITIM-receptor signaling profoundly destabilizes cytolytic synapses in resting NK cells.

## Discussion

The idea that ITIM-receptor signaling undermines NK cell-target cell interactions initially grew out of seminal experiments from Long and colleagues demonstrating that engagement of inhibitory KIRs disrupted conjugates formed by the NK cell line YTS (11). Subsequently, it was shown that stimulation of NKG2A blocked the inside-out upregulation of integrin affinity in primary NK cells (10). Single cell imaging studies with NKL cells confirmed and extended these results by demonstrating that inhibitory signals antagonized IS formation, providing a reverse stop signal that encouraged migration rather than focused cytolysis (8, 9). Here, we demonstrate that engagement of ITIM-receptors disrupts IS formation in both resting and IL-2-cultured NK cells, which is largely consistent with these previous studies.

Interestingly, whereas inhibitory stimulation promoted migration in IL-2-cultured NK cells, it induced detachment in resting NK cells. Both increased motility and membrane detachment, however, are manifestations of a destabilized IS. As we have suggested (2), the behavior exhibited by NK cells under inhibitory conditions likely depends not only on the amount of inhibitory stimulation but also on the basal levels of adhesiveness and motility in each cell type. IL-2-cultured NK cells display robust motility under non-stimulatory conditions, while resting NK cells do not. Hence, in both cases inhibitory stimulation causes the NK cells to revert to their unperturbed state, consistent with the idea that ITIM-receptors specifically target activating signals and leave other aspects of NK cell physiology intact.

Previous studies have demonstrated an important role for LFA-1-ICAM interactions in the regulation of NK cell activation. LFA-1 can drive cytoskeletal polarization toward a target cell in the absence of activating receptor stimulation (20), and it also appears to be required for establishing radially symmetric organization within the NK cell IS (21). Here, we demonstrate that LFA-1 engagement is required for IS formation and Ca^2+^ flux on supported lipid bilayers. Importantly, these activating responses could not be induced by LFA-1 alone, but only by combining LFA-1 engagement with stimulation of CD16 or NKG2D/2B4. Hence, LFA-1 appears to be behaving as an obligate costimulatory receptor in this context. These results were somewhat surprising given previous reports that stimulation of certain activating receptors, such as CD16, can drive Ca^2+^ flux in resting NK cells in the absence of LFA-1 engagement (22) (Bryceson, personal communication). In these previous studies, NK cells were activated either by antibody crosslinking in suspension or by coincubation with S2 cells expressing selected activating ligands. These differences in experimental approach could potentially explain the apparent contradiction with our work. On supported bilayers, ICAM-1 may be required for promoting close apposition of the NK cell membrane with the surface, a prerequisite for activating receptor stimulation. This sort of adhesive function would be irrelevant for antibody crosslinking experiments, and in NK cell-S2 conjugates, tight intermembrane spacing could be established by another receptor-ligand pair. Further investigation of when, where, and how LFA-1-ICAM interactions contribute to NK cell IS formation is clearly warranted.

The importance of LFA-1 for NK cell activation on bilayers might also explain the discrepancies between our results and those of (6), who found that stimulation of NKG2A did not alter IS stability. The bilayers used in this previous study contained no ICAM-1, only human Fc (to engage CD16), which based on our results would not be expected to induce bona fide IS formation on its own. Using this stimulatory regime to assess the effects of NKG2A stimulation on IS stability is therefore somewhat problematic. Recent studies have suggested that NK cells can kill targets by forming transient interactions accompanied by weak Ca^2+^ flux (23). T cell biologists have coined the term “kinapse” to describe contacts like this because they contain certain elements of activating synapses without the associated cell arrest (24). It is conceivable that the interactions visualized by Liu et al. are more akin to kinapses, and as such they are not strictly comparable to the stable, radially symmetric contacts documented here. Moving forward, it may be worthwhile for the field to establish certain objective criteria, based on architecture, stability, or associated signaling, for what qualifies as an “immunological synapse.” This could facilitate the reconciliation of observations made under different experimental conditions. Our present results, however, when taken together with previous studies, leave little doubt that inhibitory contacts are substantially less stable than activating synapses, and that ITIM-receptors induce this destabilization.

## Materials and Methods

### NK cells

Human NK cells were isolated from peripheral blood of a healthy donor by negative selection (Miltenyi Biotec). The procedure typically yielded >95% CD3^−^CD56^+^ cells. KIR2DL3^+^ and NKG2A^+^ subsets were isolated from this purified population by FACS. Resting NK cells were resuspended in CellGenix GMP SCGM medium supplemented with 10% human serum and were used within 2 days. IL-2-cultured NK cells were prepared by culturing purified resting NK cells in CellGenix GMP SCGM medium supplemented with 500 IU/ml IL-2 for at least 2 weeks. IL-2-cultured NK cells were used for experiments between 14 and 23 days after initial isolation.

### Lipid bilayers

Supported lipid bilayers containing a 10:1 mixture of DOPC and biotinyl cap phosphoethanolamine (both obtained from Avanti Polar Lipids) were prepared in eight-well chamber slides (Fisher) as described previously (8). ULBP3 (a.a. 24–207), human CD48 (a.a. 27–219), mouse ICAM-1 (a.a. 28–485), HLA-Cw3 (a.a. 25–302), and HLA-E (a.a. 22–296) were expressed with C-terminal BirA recognition sequences to enable site specific biotinylation. Recombinant proteins were purified, refolded, and biotinylated as described previously (8, 25). Biotinylated proteins were incorporated into the bilayers at the following concentrations: ULBP3 (2 μg/ml), CD48 (2 μg/ml) ICAM-1 (1 μg/ml), anti-CD16 (3G8, BD Biosciences, 2 μg/ml), human IgG1 (Jackson ImmunoResearch, 2 μg/ml), HLA-Cw3 (1 μg/ml), and HLA-E (1 μg/ml). In cases where one or more proteins were left out, a non-stimulatory biotinylated mouse MHC molecule (either I-E^k^ or H-2D^b^) was added to keep the total protein concentration constant.

### Ca^2+^ imaging

Primary NK cells were loaded with Fura-2-AM dye and imaged on lipid bilayers using a 20× objective lens (0.75 NA, Olympus). Three positions within each well were imaged every 30 s for a total of 30 min. For each position, the average Fura-2 ratio for all cells was determined as a function of time. Values during the plateau phase of the response (8–16 min) were averaged over all positions and graphed for comparison.

### Cell motility assay

IL-2-cultured NK cells were stained with PKH26 and imaged by TIRF microscopy on lipid bilayers using a 60× objective lens (1.45 NA, Olympus). Three positions were imaged every 10 s for 25 min. Single cell motility was tracked manually. Trajectories and velocities were calculated for each cell using custom MATLAB (MathWorks) scripts.

### Cell adhesion assay

Primary resting NK cells were stained with PKH26 and seeded into wells containing stimulatory bilayers. After 10 min at 37°C, the wells were washed with pre-warmed (37°C) PBS and then fixed with 4% PFA for 5 min. The total number of cells in each well was quantified at low resolution (10× objective), excluding cells that accumulated within 800 μm of the edge of the well.

## Conflict of Interest Statement

The authors declare that the research was conducted in the absence of any commercial or financial relationships that could be construed as a potential conflict of interest.
